# Doubling the Magnetorheological Effect of Magnetic Elastomers

**DOI:** 10.1021/acspolymersau.5c00118

**Published:** 2025-11-21

**Authors:** Lukas Fischer, Andreas M. Menzel

**Affiliations:** † Institut für Physik, Otto-von-Guericke-Universität Magdeburg, Universitätsplatz 2, 39106 Magdeburg, Germany; ‡ D3 Center, The University of Osaka, 1-32 Machikaneyama, Toyonaka, Osaka 560-0043, Japan; ¶ Department of Physics, The University of Osaka, 1-1 Machikaneyama, Toyonaka, Osaka 560-0043, Japan

**Keywords:** magnetorheological effect, computational materials design, microstructure, shear modulus, Young's modulus, magnetic elastomers, structural optimization

## Abstract

One of the most important properties of soft functionalized magnetic composite materials in view of their technological potential is given by the magnetorheological effect. It describes the change in rheological properties such as the shear modulus by the application of external magnetic fields. We demonstrate how computational materials design can support in approximately doubling the magnitude of this important phenomenon for magnetic elastomers. The key is to work with two perpendicular magnetic field directions. We expect future practical relevance of our concept.

## Graphical abstract

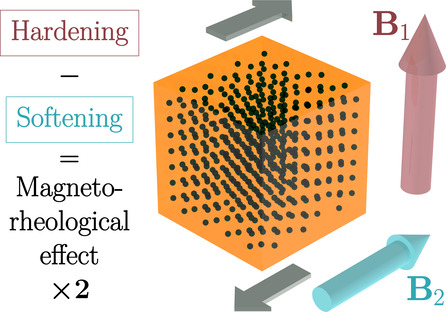

## 1 Introduction

Combining mechanical softness with functionality is intrinsic to life. We ourselves consist of soft matter that, during evolution, developed all kinds of different functional forms. It is therefore not surprising that recent research focuses on functionalizing soft materials to mimic living matter, or at least to establish corresponding compatibility.^1–3^ Examples are research on soft actuators^2,4,5^ and soft robotics.^1,2,6,7^ Often, actuation by external stimuli in a contactless manner is beneficial or mandatory. Magnetic fields stand out in this regard.^8^ They offer remote control and mostly, they are biologically harmless and do not pose any risk to health.

Both aspects are addressed and combined by magnetic elastomers. Microscopic magnetic or magnetizable particles of up to about a hundred micrometers in size are enclosed by a soft, deformable, polymeric carrier matrix. When external magnetic fields are applied, the materials respond. Specifically, they deform, which makes them promising candidates, for instance, for soft actuators^9–17^ or magnetic valves.^18,19^ Additionally, they change their overall mechanical behavior, which is referred to as the magnetorheological effect. Their stiffness, quantified in terms of their elastic Young or shear modulus, can increase by an order of magnitude.^9,20–29^ Likewise, their dissipative behavior is affected, suggesting them as tunable damping devices and vibration absorbers.^30–34^

From early attempts at structuring the arrangement of the magnetic particles inside the elastomers, we know that controlled particle positioning can significantly enhance the material properties. Specifically, during the manufacturing process, strong homogeneous magnetic fields were applied to fluid suspensions of magnetic particles in reactive solutions. Anisotropic particle aggregates formed.^13,35–42^ Then, polymerization of these structured suspensions and chemical cross-linking to form an elastomeric matrix was triggered. This process permanently locked in the anisotropic particle arrangements. When the structuring external magnetic field is turned off, the particle aggregates are maintained by the confining elastic carrier matrix. As a consequence of the established structuring, the magnetorheological effect can be enhanced substantially.^37,43^ Meanwhile, modern patterning techniques such as 3D printing,^3,44–50^ sequential photopolymerization,^51^ templated particle positioning,^52,53^ structuring by magnetic fields,^54^ acoustic holography,^55^ and wax-cast molding^56^ have been presented. Through their advent and further development, controlled positioning of magnetizable inclusions in elastomeric carrier matrices has come into reach.

Therefore, we recently addressed the question of optimizing the positioning of magnetizable inclusions in the elastic carrier matrix to maximize the requested effects.^57^ That is, we identified specific structural arrangements that most strongly enhance the magnetically induced relative changes in elastic moduli: that is, the magnetorheological effect. For this purpose, we developed a computational approach that maximizes the requested behavior as a function of the positioning of individual magnetizable sites.^57^ These magnetizable sites can consist of individual particles, magnetic droplets during 3D printing, or isotropic particle clusters. We imposed a minimum distance of these magnetic sites between each other and from the surface of the system. These gaps are filled by nonmagnetic elastomer, which we assume is reasonable in view of 3D printing. The identified spatial arrangements of magnetizable inclusions substantially enhance the magnetorheological effect compared to isotropic positioning.

Here, we proceed an additional significant step forward in our recipe of enlarging the magnetorheological effect. We understand by the magnetorheological effect generally the relative change in static elastic modulus that can maximally be achieved by the action of external magnetic fields on a given system. Specifically, we point out a way of further increasing it by approximately a factor of 2 in magnitude. Considering the relevance of contactless magnetic tuning of soft mechanical properties, we expect this basic idea to have significant technological potential.

Key to our concept is working with two perpendicular magnetic fields. We consider not only magnetically induced mechanical hardening but also mechanical softening. Both are induced on the same finalized systems but for different orientations of the magnetic field. In our recent work, we optimized the internal structural arrangement for maximized increase in mechanical stiffness.^57^ Yet, we also identified maximized decrease in mechanical stiffness.^57^ This “negative” magnetorheological effect has hardly been addressed so far.^24,58^ The basis for doubling the magnitude of the magnetorheological effect is now to combine the two antagonistic types of behavior. We optimize the arrangement of the magnetizable inclusions to facilitate both hardening and softening on the same realization of each numerical sample. Applying the magnetic field along one direction induces mechanical hardening. Choosing a perpendicular direction for the magnetic field causes mechanical softening. Consequently, controlling the direction of the magnetic field allows one to control the qualitative type of mechanical response, hardening or softening. As far as possible, we simultaneously optimize our configurations for both types of response. Switching between these two mechanical antipodes by redirecting the magnetic field implies an approximately doubled magnetorheological effect when taking the softened state as a reference and switching to the hardened state. Instead, conventional previous considerations addressed hardening under magnetization, starting from a nonmagnetized state as a reference.

## 2 Strategy and Computational Approach

To demonstrate the doubled magnetorheological effect, we proceed as follows. All of our considerations are theoretical in nature and based on computational evaluations, in the spirit of computational materials design. We focus on cubical systems. They consist of an incompressible, linearly elastic carrier medium with embedded magnetizable inclusions of vanishing magnetic remanence. Our task is to sort the magnetizable inclusions into the elastic cube in a way that maximizes the overall elastic modulus of the system when magnetized in one direction. At the same time, the overall elastic modulus should be minimized when applying the magnetic field in a perpendicular direction. Thus, the first magnetic field direction implies maximized elastic hardening/stiffening. The second, perpendicular field direction leads to maximized elastic softening.

To approach this task, we start from our previously developed computational scheme.^57^ It is based on simulated annealing^59–61^ with adaptive cooling rates.^62^ For a given number of magnetizable inclusions *N*, we optimized their positions. In such previous investigations, *either* maximized elastic hardening/stiffening *or* maximized elastic softening under the application of only one magnetic field was achieved. Thus, switching between a nonmagnetized state and a state magnetized along one direction of an external magnetic field was considered. We assumed all inclusions to be identical and magnetized to saturation when the external magnetic field is applied. Moreover, we considered affine deformations of the elastic material. The output of our computational scheme provided the spatial arrangements of the magnetizable inclusions in the cube.^57^ Generally, because of the statistical nature of the approach, which in each step suggests one discrete choice of the locations of the inclusions, and finite optimization times, this method cannot guarantee to find the fully exact global optimum. As a test, we always use eight independent optimization runs with different initial configurations. Always, they lead to very similar results. Moreover, we compare to related regular structures and always find worse performance of the latter.

Linear elasticity theory is used to characterize the incompressible elastic material and dipolar interactions to quantify the behavior of the separated magnetizable inclusions. Consequently, only one nondimensional parameter remains that determines the relative strength between these two effects. It is given by 3μ_0_ *m* ^2^/4*πμa* ^6^, where μ_0_ denotes vacuum permeability, *m* the magnetization of each inclusion in the magnetized state, μ the elastic shear modulus of the elastic component, *a* the side length of the cubical system, and we here assume monodisperse spherical inclusions of radius 0.03*a*. To choose a realistic value for the nondimensional number, we compare to possible experimental parameters of a saturation magnetization of the inclusions of 518 kA m^–1^ as for Fe_3_O_4_ ^63^ and a shear modulus of μ ≈ 1.67 kPa.^10,11,64,65^ These values lead to a nondimensional number of approximately 6.2 × 10^–7^, which we used in all cases. Increasing this nondimensional number, for example, by using a softer elastic component, increases the resulting effects in magnitude.

For our present purpose of demonstrating the doubled magnetorheological effect, we modified our computational scheme accordingly. We now optimize the spatial arrangement of the magnetizable inclusions such that the system shows a maximized difference in elastic modulus when switching an applied saturating external magnetic field between two perpendicular directions, along **B**_1_ and **B**_2_, see Figure 1. Besides modifying the optimization scheme accordingly, some operational parameters in the optimization algorithm^57^ were adjusted; see the Supporting Information for details.

**Figure 1:**
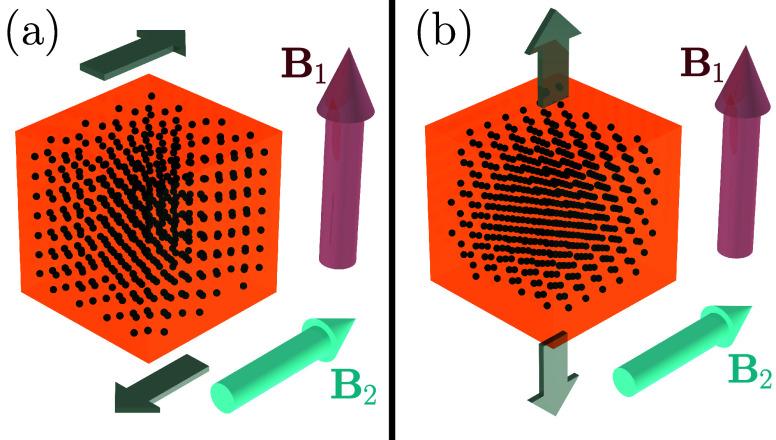
Illustration of the considered geometries. A cubical elastic system (orange) with *N* magnetizable inclusions (dark dots) is subject to an externally imposed mechanical deformation as indicated by the flat arrows. Both (a) shear and (b) uniaxial elongation are addressed. An external magnetic field is either applied along the cylindrical red arrow **B**_1_, or perpendicular to it, as marked by the cylindrical turquoise arrow **B**_2_. Each realization of the system is simultaneously optimized concerning the positions of the magnetizable inclusions for elastic hardening/stiffening under magnetic field application along one direction and elastic softening under magnetic field application along the other direction. Switching between the two magnetic field directions then implies an approximately doubled magnetorheological effect compared to the nonmagnetized situation as a state of reference.

In detail, we calculate the modified elastic modulus for each tested configuration as follows. We determine in the magnetized state the sum of the elastic and dipolar magnetic interaction energies and identify the new energetic minimum. Close to the minimum, we fit the overall energy under imposed deformation, see Figure 1, by a parabola. The resulting coefficient of the parabolic fit is identified as the new elastic modulus. This modulus should be maximally increased to μ_hard_ when compared with the nonmagnetized state when magnetizing the system along one direction. Simultaneously, it should be maximally decreased to μ_soft_ when magnetizing along a perpendicular direction. Two perpendicular external magnetic fields **B**_1_ and **B**_2_ are considered to this end, applied in turns but not simultaneously. We consider them close to saturating, so that effects of magnetic remanence can be neglected below.

The resulting overall magnetorheological (MR) effect is then defined as the relative change in the elastic modulus when switching between these two cases. If we require maximized hardening/stiffening when applying **B**_1_ and take the softened state under **B**_2_ as an initial state, we identify the MR effect with (μ_hard_ – μ_soft_)/μ. Conversely, if we aim for maximized softening when applying **B**_1_ and take the hardened/stiffened state under **B**_2_ as an initial state, we define the MR effect as (μ_soft_ – μ_hard_)/μ. For a better quantitative comparison between the two directions of changing the modulus, we always evaluate it relative to the elastic modulus μ of the (nonmagnetized) elastic matrix material. (A remark at the end of the Supporting Information adds more to this point.)

To illustrate the doubled magnetorheological effect, we focus on two geometries; see Figure 1. The two perpendicular magnetic field directions **B**_1_ and **B**_2_ follow the edge orientations of the cube. First, we consider imposed shear deformations of the cubical system; see Figure 1(a). The shear displacements are imposed parallel to **B**_2_, while the gradient of the shear displacements aligns with **B**_1_. Second, we address uniaxial stretching, as illustrated in Figure 1(b). Lateral contraction follows from the assumed incompressibility of the material. Here, one magnetic field direction **B**_1_ is along the stretching axis. The other magnetic field direction **B**_2_ is perpendicular to the stretching axis.

## 3 Results and Discussion

We start with the case of externally imposed shear deformations, as displayed in Figure 1(a). To achieve the maximized overall change in shear modulus, we optimize the spatial configuration of the magnetizable inclusions to maximize elastic hardening/stiffening under application of the magnetic field **B**_1_, while simultaneously optimizing for maximized elastic softening under application of the perpendicular magnetic field **B**_2_. The procedure is performed for different fixed numbers of magnetic inclusions *N*. Figure 2 displays by the red curve the resulting relative change in elastic shear modulus when switching between the two perpendicular magnetic field directions, **B**_1_ and **B**_2_. As expected, the magnetically induced relative change in shear modulus increases with an increasing number of inclusions *N*. For comparison, we also include our previous results^57^ optimized only for elastic hardening under application of the magnetic field **B**_1_, with the reference state set as the nonmagnetized system, see the blue curve.

**Figure 2:**
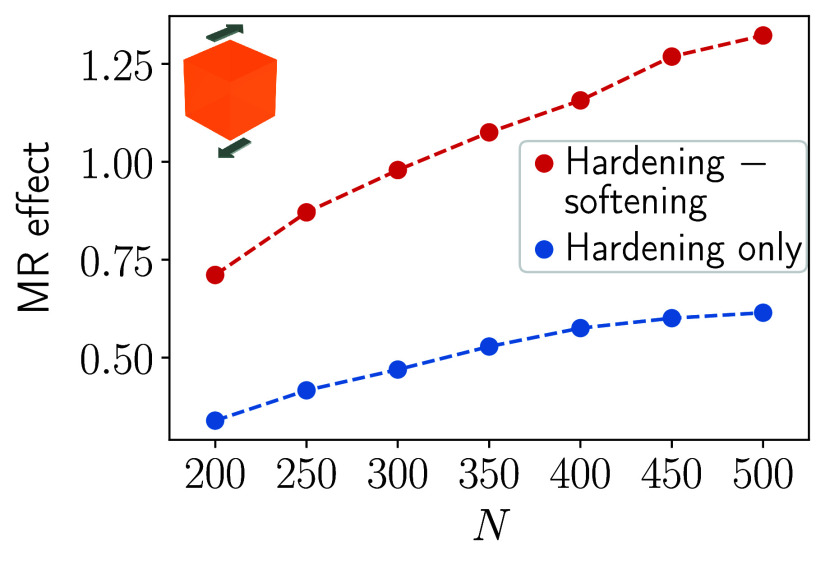
Magnitude of the magnetorheological effect (MR effect) under imposed shear deformation, see the geometry in Figure 1(a), as a function of the number of magnetizable inclusions *N*. The MR effect is defined as the relative change in overall elastic shear modulus when switching to the mechanically hardened/stiffened state, see Section 2. We switch between two perpendicular magnetic fields (red curve); see Figure 1(a). One field triggers maximized mechanical hardening/stiffening, the other one induces maximized mechanical softening. The resulting relative change in elastic modulus is more than doubled in magnitude when compared to previous situations optimized for hardening/stiffening only that solely used the external magnetic field **B**_1_ (blue curve).^57^

We can infer that, as a consequence of the bidirectional optimization strategy and switching between the two perpendicular magnetic fields, we have more than doubled the magnetorheological effect by comparing the red and the blue curves. For the highest considered number of inclusions *N* = 500, the magnetically induced relative change in elastic shear modulus (red curve) is approximately of a factor 2.15 larger when compared to the previously observed, already maximized effect working with only one magnetic field direction (blue curve). We include Supporting Information concerning the spatial arrangement of the magnetizable inclusions in the optimized configuration.

Next, we consider the opposite switching scenario. That is, we optimize the spatial configuration of the magnetizable inclusions for maximized elastic softening when magnetic field **B**_1_ is applied. Simultaneously, we optimize for maximized elastic hardening/stiffening under application of the magnetic field **B**_2_. The results are displayed in Figure 3. Here, the magnitude of the induced relative change in elastic modulus is a bit less than doubled (red curve) when compared to the previously optimized scenario that only considers magnetically induced mechanical softening relative to the nonmagnetized state of the system (blue curve). Since mechanical softening is associated with a negative change in the magnitude of the elastic shear modulus, this scenario was termed negative magnetorheological effect.^24,58^ For the highest considered number of magnetizable inclusions *N* = 500, the relative change in elastic shear modulus in Figure 3 is approximately by a factor of 1.80 larger (red curve) than the previously maximized value (blue curve).

**Figure 3:**
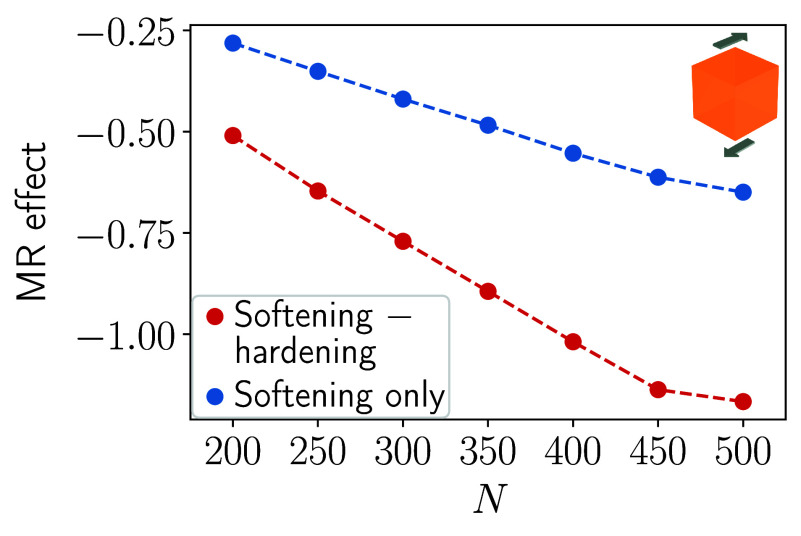
Similar to Figure 2, but now maximized mechanical softening induced by **B**_1_ and maximized elastic hardening/stiffening caused by **B**_2_. The relative change in elastic shear modulus (MR effect) for mechanical softening is negative, see the sign on the vertical axis. Again, the induced effect when working with two perpendicular magnetic field directions (red curve) is approximately doubled when compared to the previous optimization scheme^57^ of using only **B**_1_ to induce elastic softening (blue curve).

In a second step, we consider imposed uniaxial stretching, as indicated in Figure 1(b). Here, we evaluate the magnetically induced relative change in elastic Young/stretching modulus. We simultaneously optimize the spatial arrangement of the magnetizable inclusions for maximized mechanical hardening/stiffening under application of the magnetic field **B**_1_ and for maximized mechanical softening under the perpendicular magnetic field **B**_2_. Corresponding results are displayed by the red curve in Figure 4 when switching between both fields. Again, the relative change in elastic Young modulus is substantially enlarged (red curve) when compared to previous results that only use one magnetic field and solely maximize for elastic hardening (blue curve).^57^ For the number of magnetizable inclusions *N* = 500, we here observe an increase by a factor of approximately 1.62.

**Figure 4:**
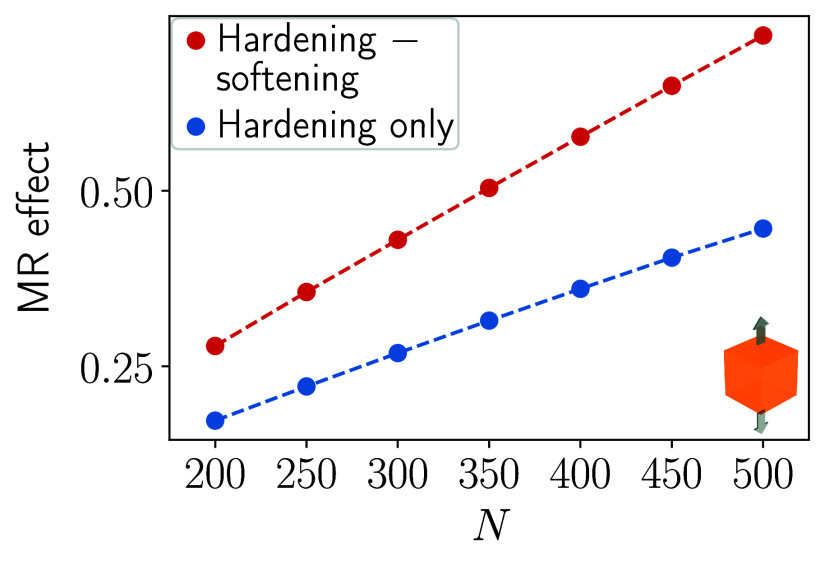
Analogous to Figure 2, but now for the relative change in elastic Young/stretching modulus (see Figure 1(b)) when switching from **B**_2_ to **B**_1_ (red curve). We also include our previous results obtained by maximizing solely for elastic hardening/stiffening when turning on **B**_1_ from zero (blue curve).^57^

Again, we address the inverted situation as well. That is, we optimize for maximized elastic softening under application of the magnetic field **B**_1_, while simultaneously optimizing for maximized elastic hardening/stiffening under the perpendicular magnetic field **B**_2_. Figure 5 displays the corresponding results when switching from **B**_2_ to **B**_1_ (red curve). Once more, this strategy of using two perpendicular external magnetic fields for correspondingly optimized spatial arrangements leads to significantly enhanced effects when compared to the previous situation of using only **B**_1_ to soften the material from the nonmagnetized state (blue curve).^57^ The maximal relative change in Young modulus for the number of magnetizable inclusions *N* = 500 was about a factor of 1.57 larger (red curve) when compared to the previous results of optimizing the structure for switching from the nonmagnetized state (blue curve).^57^ Due to the considered mechanical softening, the sign of the change in elastic modulus in Figure 5 is negative.

**Figure 5:**
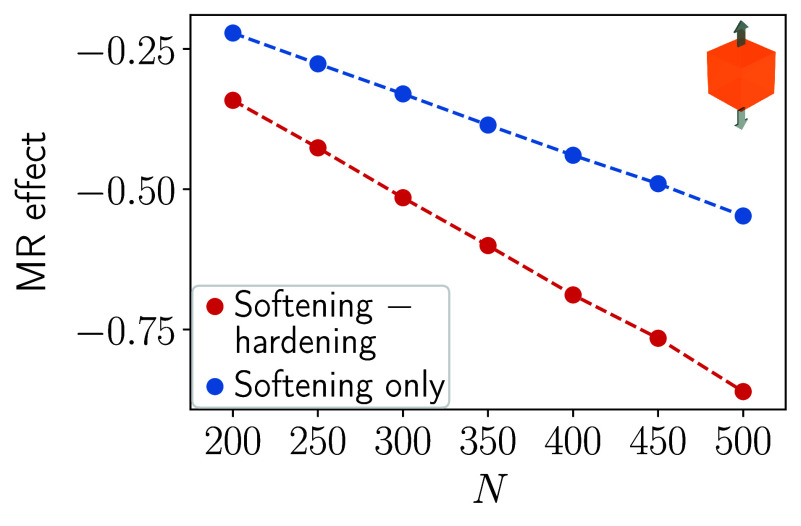
Similar to Figure 4 for the elastic Young/stretching modulus, but now inducing maximized mechanical softening by the magnetic field **B**_1_ and maximized mechanical hardening/stiffening under the perpendicular magnetic field **B**_2_. We reveal significantly enhanced changes in elastic Young/stretching modulus when switching between the two perpendicular magnetic fields **B**_1_ and **B**_2_ for the correspondingly optimized structures (red curve). They are compared to previous data for maximized softening only when solely turning on **B**_1_ from zero (blue curve).^57^

Additional information on the resulting configurations of the magnetizable inclusions for the four different situations studied above is included in the Supporting Information. In the last situation of uniaxial stretching, we can directly infer the influence of the second perpendicular magnetic field when performing the optimization for the alternating action of the two perpendicular magnetic fields. If optimization is solely performed for the action of **B**_1_ along the stretching axis, cubical symmetry in the plane perpendicular to the stretching axis is approximately maintained in resulting configurations. However, simultaneously considering the alternately applied perpendicular magnetic field **B**_2_ in the optimization scheme breaks this transversal cubical symmetry, which is reflected by the spatial organization of the magnetizable inclusions. Besides, we checked that the performance for the response solely to the magnetic field **B**_1_, turning this field on from the nonmagnetized state, for our bidirectionally optimized systems, is only slightly lower when compared to our previous results specifically optimized for just one magnetic field **B**_1_.^57^

## 4 Conclusions

In conclusion, we outline here a path of designing magnetorheological materials that feature a substantially enhanced magnetically induced relative change in mechanical properties. Our strategy is based on positioning the magnetizable inclusions in a soft, elastic carrier medium in a way that is most beneficial for the desired purpose. Already in a previous investigation, we optimized the spatial arrangement of the magnetizable inclusions in a corresponding manner.^57^ Yet, there we still considered the nonmagnetized states of the systems as the states of reference to quantify the magnetically induced relative change in mechanical properties. Generally, this has been the conventional point of reference so far. The structures were thus optimized for switching from a nonmagnetized to a magnetized state.

Equipped with our modified computational formalism, we here employ switching between two perpendicular magnetic fields. The spatial arrangement of the magnetizable inclusions is optimized in a way that one magnetic field direction induces maximized elastic hardening. The other magnetic field direction implies maximized elastic softening. In combination, when switching between these two perpendicular magnetic fields, the magnitude of induced relative change in elastic moduli is approximately doubled when compared to the situation that employs only one magnetic field direction.

Not necessarily are we thinking of rigid magnetizable inclusions, but also droplets of magnetic fluid could be considered in reality, for instance, during 3D printing processes.^45,50^ This should be helpful when our suggestions of material design are transferred to reality. Soft magnetic elastomers have a large potential when it comes to magnetically tuning mechanical properties. Promoting an approximate doubling in the magnitude of these effects should raise enhanced technological interest in these materials. Generally, as a strategy, one can work from lower to higher volume fractions of magnetizable inclusions, both in computational design and in experimental realization.

## Supplementary Material



## Notes

Parameter settings in the employed optimization algorithm; information on the structures of the optimized configurations; discussion of a shift in numbers when modifying the definition of the MR effect (PDF)
