# Supplementary material for: Doubling the Magnetorheological Effect of Magnetic Elastomers
Source: ACS Polym Au. 2025 Nov 21;6(1):157–63. doi: 10.1021/acspolymersau.5c00118 (PMC12903475; doi:10.1021/acspolymersau.5c00118)
Supplement: Supplementary file 1 [file lg5c00118_si_001.pdf]

# Supporting Information
# "Doubling the magnetorheological effect of magnetic elastomers"

Lukas Fischer[1, 2, 3, *] and Andreas M. Menzel[1, †]

[1] *Institut für Physik, Otto-von-Guericke-Universität Magdeburg, Universitätsplatz 2, 39106 Magdeburg, Germany*
[2] *D3 Center, The University of Osaka, 1-32 Machikaneyama, Toyonaka, Osaka 560-0043, Japan*
[3] *Department of Physics, The University of Osaka, 1-1 Machikaneyama, Toyonaka, Osaka 560-0043, Japan*

(Dated: October 22, 2025)

In the main text, we describe how optimized arrangements of magnetizable inclusions lead to maximized magnetorheological effects under switching of external magnetic fields between mutually perpendicular orientations. Here, we first summarize details and parameters that were used for our optimization procedure. Afterwards, we include additional information about these optimized configurations.

## DETAILS ON THE OPTIMIZATION METHOD

Our optimization method for the MR effect relative to a nonmagnetized base state was outlined in Ref. 1, see the supplementary material of this reference. Here, we use similar parameters for the optimization. Still, the optimized quantity is the magnitude of the MR effect as defined in the main text, which is replacing $\Delta\mu_{rel}$ in our previous optimization. We set the parameters (see the supplementary material of Ref. 1 for the definitions): $\delta = 0.03\,a$, $n_{jump} = 100$, $T_i = 3.2\times10^{-4}$, $N_{eq} = 2\,N$, $N_{prod} = 3\,N$, $N_{cool} = 25\,N$, $k_s = 2\times10^{-9}$, $k_f = 4\times10^{-9}$, $C_V^* = 1$, $patience = 20$, and $T_f = 1.36\times10^{-6}$. When changing the number of magnetizable inclusions $N$, we follow the same procedure for adjusting the parameters as outlined in the supplementary material of Ref. 1, see the following table, similar to Table VIII in the supplementary material of Ref. 1:

| $N$ | $\alpha$ | initialization |
|---|---|---|
| 200 | 1 | random |
| 250 | 1.25 | random |
| 300 | 1.5 | random |
| 350 | 1.75 | random |
| 400 | 2 | hexagonal |
| 450 | 2.25 | hexagonal |
| 500 | 2.5 | hexagonal |

TABLE I. Parameter settings in our SA algorithm when changing the number of magnetizable inclusions $N$.

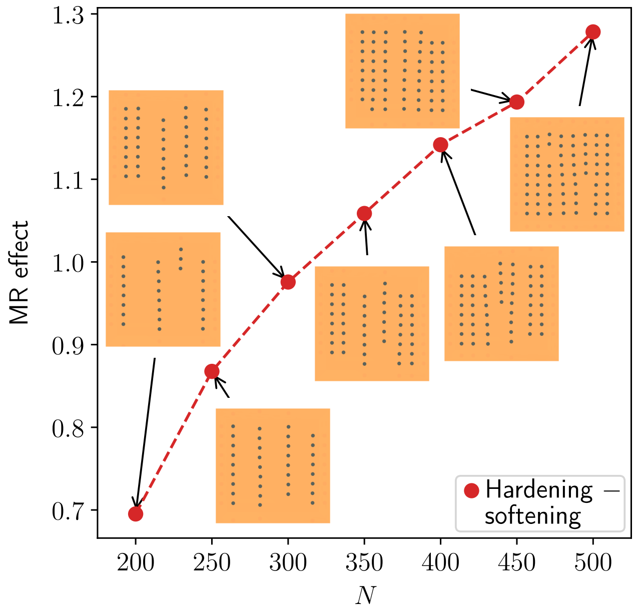


FIG. S1. Same as the red curve in Fig. 2 of the main article, indicating the increase in magnitude of the magnetorheological (MR) effect with increasing number of magnetizable inclusions $N$. Here, as insets, top views of the optimized configurations for doubling the magnetorheological effect are added, as viewed along the direction of the magnetic field $\mathbf{B}_1$, see Fig. 1 in the main article. Each dot in the snapshots marks the location of a chain-like aggregate extending below the dot, oriented along $\mathbf{B}_1$. Both the magnetic field $\mathbf{B}_2$ and the displacements under the imposed shear deformations that are considered in this configuration are oriented horizontally.

## OPTIMIZED ARRANGEMENTS FOR THE MAGNETORHEOLOGICAL EFFECTS

We analyze the identified configurations that lead to the optimized MR effects as presented in the main text. Specifically, we discuss how the full arrangement of $N$ magnetizable inclusions can be approximated by building blocks such as chains (along a certain direction) or lattice-type arrangements.

As in the main text, we start by discussing the arrangements that lead to the largest increase of shear modulus when applying the external magnetic field $\mathbf{B}_1$, see Fig. 1(a) in the main text, while applying the magnetic field $\mathbf{B}_2$ leads to maximized softening.

The insets in Fig. S1 show associated configurations in top views, that is, along the direction of the magnetic field $\mathbf{B}_1$. Each dot marks a chain-like aggregate as

viewed from the top. As the lines of dots in the insets indicate, keeping in mind the underlying chain-like aggregates, the magnetizable inclusions are thus organized in layers. The normals of these layers point into the direction of the shear displacements (parallel to the direction of the magnetic field $\mathbf{B}_2$). Within each layer, the chains are shifted relative to their neighbors along the direction of $\mathbf{B}_1$. As a consequence, the organization of the inclusions within each layer is approximately hexagonal. Similar results have been found in our previous investigation [1], where we only optimized for hardening of the system when applying $\mathbf{B}_1$, and not simultaneously for softening when applying $\mathbf{B}_2$.

Increasing the number of magnetizable inclusions $N$, the spacing between the layers is reduced to accommodate more inclusions, see the insets in Fig. S1. Thus, qualitatively, optimizing simultaneously for the potential application of the magnetic field along either of the two perpendicular directions leads to similar configurations as for our previous case of only applying the field $\mathbf{B}_1$.

Next, we address the opposite combination, namely, maximized softening when applying the magnetic field $\mathbf{B}_1$ and maximized hardening when applying the field $\mathbf{B}_2$. Again, the identified final configuration resembles layers of magnetizable inclusions of internal hexagonal positional order. However, the orientation of these layers is different. They now coincide with the shear planes, that is, they are parallel to the plane spanned by the two external magnetic fields. The chain-like aggregates forming each layer are now oriented approximately parallel to $\mathbf{B}_2$.

To test our assertion of an approximate layerwise hexagonal arrangement of the optimized configuration, we constructed such configurations by hand for comparison. Results for the MR effect of these configurations arranged by hand but with parameters extracted from the optimization procedure are compared to those of the computational output in Fig. S2. As the figure demonstrates, the performance of the reconstructed hexagonal layer-like configurations follow the same trend as the optimized configurations. Still, the latter perform significantly better (by about 20–30 %). Obviously, the irregularities in the optimized configurations that deviate from the regular structures constructed by hand cause a significant increase in performance.

Next, we turn to the MR effect induced by application of the saturating magnetic fields $\mathbf{B}_1$ and $\mathbf{B}_2$ under imposed uniaxial stretching, see Fig. 1(b) in the main article. First, we again optimize for hardening when applying $\mathbf{B}_1$, while we optimize for softening when switching to $\mathbf{B}_2$. Results in the main article are summarized in Fig. 4. In this case, the structure resulting from computational optimization resembles a face-centered cubic (fcc) lattice arrangement. Two of the cubic axes are oriented parallel to $\mathbf{B}_1$ and $\mathbf{B}_2$.

Thus, for comparison, we here constructed by hand corresponding regular fcc configurations using parameters as extracted from the computational results. Figure S3 shows that both, the curves resulting from computational optimization and from reconstruction by hand, feature qualitatively similar trends. Yet, again, the structures directly resulting from the optimization procedure perform stronger, here by 6–15 % in magnitude.

Finally, we again consider uniaxial stretching along $\mathbf{B}_1$, yet optimizing for softening when applying $\mathbf{B}_1$ and for hardening when applying $\mathbf{B}_2$. Here, we once more found that the associated optimized configurations mainly consist of chain-like aggregates oriented along the stretching

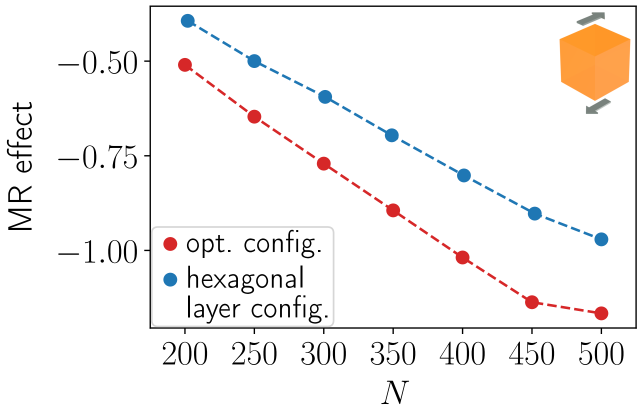


FIG. S2. The red line is identical to the one in Fig. 3 of the main article and results from computational optimization for softening or hardening when $\mathbf{B}_1$ or $\mathbf{B}_2$ is applied (“opt. config.”). Conversely, the blue line results when mimicking these structures by regular hexagonal layer-like arrangements (“hexagonal layer config.”), which we constructed by hand for comparison. Both curves follow the same trend. Yet, the configurations resulting from optimization indicate enhanced performance.

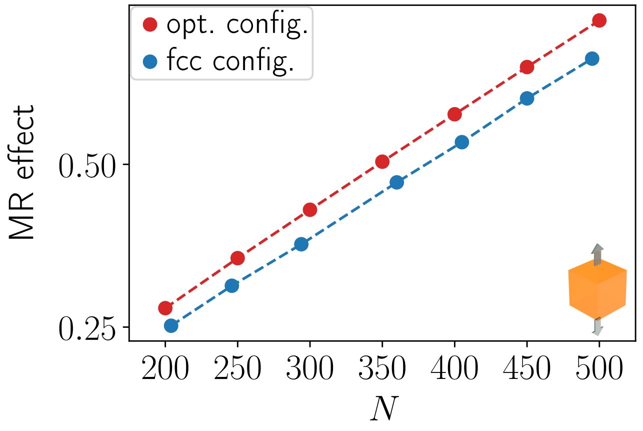


FIG. S3. Same as in Fig. S2, yet for uniaxial stretching along $\mathbf{B}_1$, as in Fig. 4 of the main article. We compare the MR effect resulting for the optimized arrangements to that of a reconstructed regular face-centered cubic (fcc) configuration.

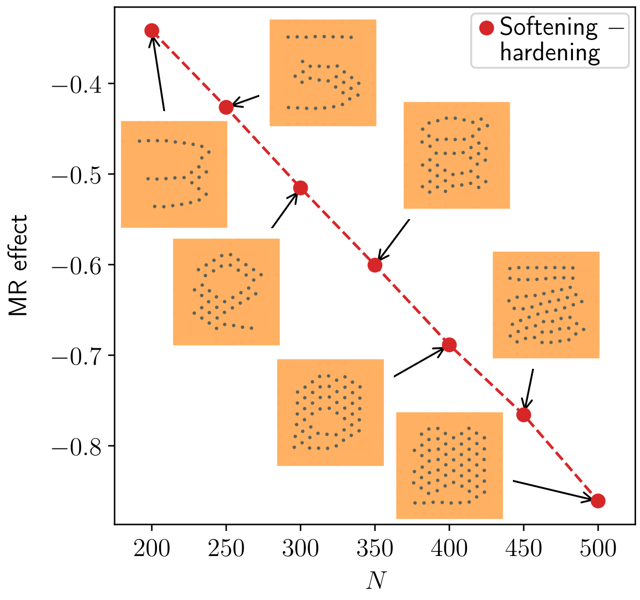


FIG. S4. Same as in Fig. S1, but for the MR effect under imposed uniaxial stretching along $\mathbf{B}_1$, which corresponds to Fig. 5 in the main article. Here, $\mathbf{B}_1$ induces mechanical softening during stretching and $\mathbf{B}_2$ mechanical hardening. The insets represent top views of the optimized configurations, viewed along the stretching axis and $\mathbf{B}_1$. Each dot implies an underlying chain-like aggregate. We clearly see how the additional and simultaneous optimization with respect to $\mathbf{B}_2$, oriented horizontally in the insets, leads to additional anisotropy of the structures, specifically at lower values of $N$.

axis. In the insets of Fig. S4, each dot marks the top view on one such chain-like aggregate.

When we optimize only for softening due to $\mathbf{B}_1$, ignoring hardening under $\mathbf{B}_2$, we find approximate isotropy of the configurations within the planes normal to the stretching axis [1]. To some degree, this isotropy is broken by the overall cubical shape of the system. Now performing the optimization when also taking into account the external magnetic field $\mathbf{B}_2$, this cubic symmetry is broken. Indeed, we now observe additional, anisotropic organization of the inclusions perpendicular to the stretching axis. In the top views shown as insets of Fig. S1, this anisotropy becomes apparent. There, simultaneous optimization for possible hardening under $\mathbf{B}_2$ leads to additional chain-like organization along $\mathbf{B}_2$, here oriented horizontally. The effect is particularly obvious for lower numbers of inclusions $N$.

## ALTERNATIVE DEFINITION OF THE MAGNETORHEOLOGICAL EFFECT

In the main article, we define the magnetorheological effect as $(\mu_{\mathrm{hard}} - \mu_{\mathrm{soft}})/\mu$ for the hardening scenario and $(\mu_{\mathrm{soft}} - \mu_{\mathrm{hard}})/\mu$ for softening. In both cases, we normalize the magnetically induced switching in elastic modulus by the elastic modulus $\mu$ of the matrix material, which here corresponds to the elastic modulus of the nonmagnetized system. The reason for these definitions using $\mu$ in the denominators in both cases was to make better comparable in magnitude the two opposite scenarios of hardening and softening.

Naturally, we can also plot the resulting MR effect for division by the elastic modulus of the initial state of the system. This leads to a shift in absolute numbers. As an example, we here focus on the case of hardening under imposed shear deformation. In Fig. S5, we replot for comparison the same data as in Fig. 2 of the main article. They result from the definition of the MR effect according to $(\mu_{\mathrm{hard}} - \mu_{\mathrm{soft}})/\mu$ (blue curve). Now, we also plot the data when changing this definition to $(\mu_{\mathrm{hard}} - \mu_{\mathrm{soft}})/\mu_{\mathrm{soft}}$ (red curve). Obviously, this redefinition here leads to a significantly increased magnitude. For the largest considered number of magnetizable inclusions $N = 500$, the relative change in elastic shear modulus (according to the new definition) in Fig. S5 is approximately by a factor of 3.60 larger (red curve) than the value in Fig. 2 of the main article (blue curve).

* fischer.lukas.d3c@osaka-u.ac.jp
† a.menzel@ovgu.de
[1] L. Fischer and A. M. Menzel, Maximized response by structural optimization of soft elastic composite systems, PNAS Nexus **3**, pgae353 (2024).

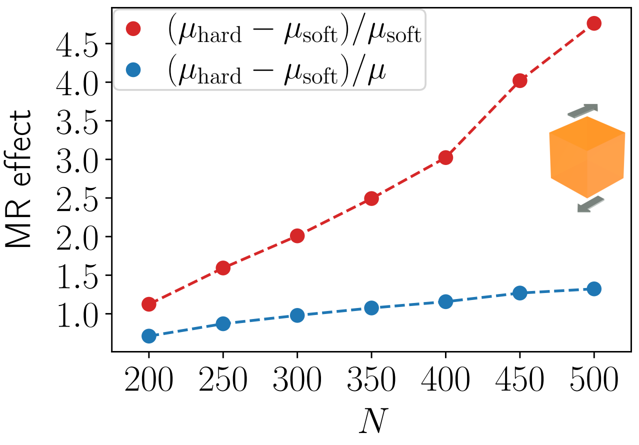


FIG. S5. Same as Fig. 2 of the main article when we redefine the MR effect as $(\mu_{\mathrm{hard}} - \mu_{\mathrm{soft}})/\mu_{\mathrm{soft}}$ (red curve) instead of $(\mu_{\mathrm{hard}} - \mu_{\mathrm{soft}})/\mu$ (blue curve). The different definition leads to significantly larger numbers.
